# Veterinary antimicrobials in cattle feedlot environs and irrigation conveyances in a high-intensity agroecosystem in southern Alberta, Canada

**DOI:** 10.1007/s11356-022-22889-x

**Published:** 2022-09-15

**Authors:** Srinivas Sura, Francis J. Larney, Jollin Charest, Tim A. McAllister, John V. Headley, Allan J. Cessna

**Affiliations:** 1 Agriculture and Agri-Food Canada, Morden Research and Development Centre, 101 Route 100, Morden, MB R6M 1Y5 Canada; 2 Agriculture and Agri-Food Canada, Lethbridge Research and Development Centre, 5403 1st Avenue S, Lethbridge, AB T1J 4B1 Canada; 3 Natural Resource Management Branch, Alberta Agriculture, Forestry, and Rural Economic Development, 5401 1st Avenue S, Lethbridge, AB T1J 4V6 Canada; 4 Environment and Climate Change Canada, National Hydrology Research Centre, 11 Innovation Blvd, Saskatoon, SK S7N 3H5 Canada; 5 Agriculture and Agri-Food Canada, Saskatoon Research and Development Centre, 107 Science Place, Saskatoon, SK S7N 0X2 Canada

**Keywords:** Veterinary antimicrobial, Surface water, Beef cattle, Manure, Irrigation, Intensive agroecosystem, Alberta

## Abstract

The South Saskatchewan River Basin (SSRB) is considered one of the most intensively farmed regions in Canada, with high densities of livestock and expansive areas of irrigated cropland. We measured concentrations of seven veterinary antimicrobials (VAs) in 114 surface water samples from feedlot environs and 219 samples from irrigation conveyances in the SSRB. Overall, detection frequencies in feedlot environs were 100% for chlortetracycline (CTC) and tetracycline (TC), 94% for monensin (MON), 84% for tylosin (TYL), 72% for lincomycin (LIN), 66% for erythromycin (ERY), and 23% for sulfamethazine (SMZ). For irrigation conveyances, detection frequencies for CTC and TC remained high (94–100%), but dropped to 18% for ERY, 15% for TYL, 10% for MON, and 4% for SMZ. Lincomycin was not detected in irrigation conveyance water. Maximum concentrations of VAs ranged from 1384 µg L^−1^ (TC) to 17 ng L^−1^ (SMZ) in feedlot environs while those in irrigation conveyances were 155 ng L^−1^ (TC) to 29 ng L^−1^ (ERY). High detection frequencies and median concentrations of VAs in both feedlot environs and irrigation conveyances were associated with high amounts of precipitation. However, an irrigation district (ID) with high livestock density (Lethbridge Northern) did not exhibit higher concentrations of VAs compared to IDs with less livestock, while levels of VAs in irrigation conveyances were less influenced by the degree of surface runoff. The ubiquity of CTC and TC in our study is likely a reflection of its widespread use in intensive livestock operations. Additional investigation is required to link environmental concentrations of VAs with livestock densities and increase our understanding of potential antimicrobial resistance in high-intensity agroecosystems.

**Supplementary Information:**

The online version contains supplementary material available at 10.1007/s11356-022-22889-x.

## Introduction

**Fig. 1:**
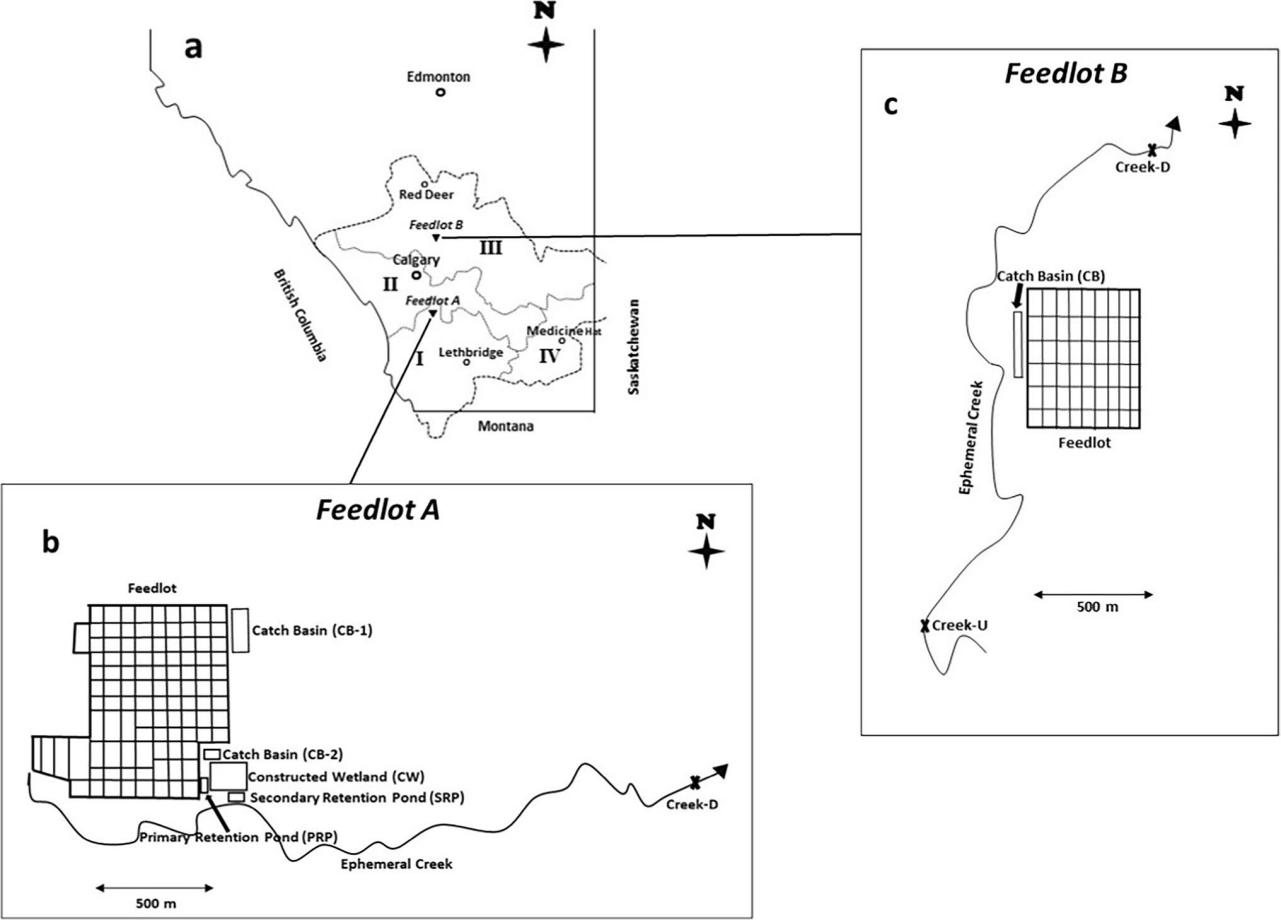
**a** Southern Alberta showing the South Saskatchewan River Basin comprising (I) Oldman River, (II) Bow River, (III) Red Deer River, and (IV) South Saskatchewan River sub-basins and relative locations of Feedlots A and B; **b** Feedlot A showing 6 water sampling sites: catch basin 1 (CB-1), catch basin 2 (CB-2), primary retention pond (PRP), constructed wetland (CW), secondary retention pond (SRP), and downstream creek (Creek-D); **c** feedlot B showing 3 water sampling sites: catch basin (CB), downstream creek (Creek-D), and upstream creek (Creek-U)

In southern Alberta, the South Saskatchewan River Basin (SSRB) comprises the Oldman, Bow, Red Deer, and a portion of the South Saskatchewan River sub-basins (Fig. 1a). The rivers generally flow eastward from the Rocky Mountains, through the foothills and prairie, with a combined watershed area of 121,000 km^2^ in Alberta. The climate is semiarid with annual precipitation ranging from 900 mm in the sub-alpine west to 300 mm in mixed grassland in the east. Except for the upper reaches in the mountains and foothills, the SSRB is considered one of the most intensively farmed regions in Canada due to high densities of beef cattle (grazing, confined feeding operations), dairy, swine, and poultry; and the largest area of irrigated cropland in the country (Schindler and Donahue 2006; Alberta Environment 2006; Alberta Agriculture and Forestry 2021).

In 2015, Alberta had 5.2 million head of cattle (Statistics Canada 2016), or 42% of Canada’s national cattle herd, and ranked fourth in North America, after Texas (11.7 million), Nebraska (6.5 million), and Kansas (6.3 million) [USDA 2020]. In addition, Alberta had 1.4 million swine and 14.9 million poultry. The majority of Alberta’s intensive livestock industry (i.e., confined feeding operations, including beef cattle feedlots) is located in the SSRB, largely due to access to irrigation for the production of livestock feed (silage, hay, forage, grain) and a favorable climate. In recent decades, Lethbridge County, in the Oldman sub-basin of the SSRB, has had the highest number of animal units (e.g., 427,000 in 2001) in Canada (Beaulieu and Bédard 2003).

Veterinary antimicrobials (VAs) are widely administered to livestock, therapeutically for treatment of infection, and sub-therapeutically for disease prevention (Sarmah et al. 2006). The estimated average VAs sold in Canada in 2019 per population correction unit (PCU) were at 73 mg/PCU for cattle, 175 mg/PCU for poultry, and 278 mg/PCU for swine (Government of Canada 2022). Excretion rates in manure (feces and urine) may approach 95% (Kim et al. 2011), depending on the VA, its dose, growth stage, and species of livestock (Zhou et al. 2013). The presence of VAs in aquatic environments depends not only on their physico-chemical properties but also on veterinary and agricultural practices, climate conditions, and landscape characteristics such as soil type, slope, and buffer zones (Jaffrézic et al. 2017). Land application of manure is a major point of entry of VAs to the broader agroecosystem, leading to (i) contamination of surface and groundwater (Kümmerer 2009; Lapworth et al. 2012), (ii) potential uptake by crops destined for human consumption (Azanu et al. 2016; Tasho and Cho 2016), and (iii) selection for pathogenic bacteria harboring antimicrobial resistance genes (Heuer et al. 2011; Chattopadhyay 2014), which may reduce the therapeutic efficacy of antimicrobials against bacterial infections in humans and animals (Landers et al. 2012). From 1981 to 2001, seven of the ten sub-sub-drainage areas showing the largest increases (1.6–4.1 Mg ha^−1^) in manure production in Canada were in the SSRB (Statistics Canada 2006). Cattle (beef, dairy) generated > 90% of the manure, with lesser amounts from swine and poultry.

In 2015, Alberta had 28% of Canada’s total cropland and 71% of Canada’s irrigated cropland (Statistics Canada 2016). Land use intensity and input requirements are higher under irrigation than in dryland farming, due to higher yields, greater crop diversification, and avoidance of drought (Irrigation Water Management Study Committee 2002). Almost 98% of Alberta’s irrigation occurs within the SSRB (Paterson Earth & Water Consulting 2015), most of which (566,000 ha) is managed by about 6000 farmers within 13 irrigation districts (ID). Irrigation within the SSRB relies on surface water from spring snowmelt in the Rocky Mountains which is stored in on-stream and off-stream reservoirs, with a total capacity of ~ 3 billion m^3^, and delivered through ~ 8000 km of conveyance networks of canals and pipelines (Paterson Earth & Water Consulting, 2015). Water is supplied not only to irrigated crops, but also to livestock, rural residents for household use, and municipalities for swimming pools, parks, and industrial use, including food processing. Water stored in irrigation reservoirs provides wildlife habitat and recreational opportunities such as fishing, boating, and camping (Irrigation Water Management Study Committee 2002).

The occurrence of VAs in aquatic environments (surface and groundwater) has been widely reported in Canada (Couperus et al. 2016; Kleywegt et al. 2011; Schwartz et al. 2021) and globally (Alonso et al. 2019; Danner et al. 2019; Felis et al. 2020). In an analysis of 247 water samples from 23 Alberta watersheds, Forrest et al. (2011) detected chlortetracycline (CTC), sulfamethazine (SMZ), monensin (MON), lincomycin (LIN), erythromycin (ERY), and other VAs. In southern Alberta, Sura et al. (2015) reported maximum runoff estimates based on a simulation of a one in a 100-year rainfall event of 1.3–3.6 g head^−1^ of CTC, 1.9 g head^−1^ of SMZ, and 0.2 g head^−1^ of tylosin (TYL), to catch basins from beef cattle feedlot pens. After land application of feedlot manure in southern Alberta, Amarakoon et al. (2014) reported mass losses in surface runoff of CTC > SMZ > TYL (expressed as a percent of amounts applied), which were independent of their respective soil sorption coefficients (*K*d values). Moreover, Amarakoon et al. (2016) measured CTC in soil 10 months after manure application and found that there was a potential risk for the build-up of VA residues if feedlot manure was repeatedly applied to the same land. Kuchta et al. (2009) showed that LIN in manure can persist in the environment for several months and percolate into groundwater. Furthermore, Leung et al. (2013) reported median concentrations of 10 ng L^−1^ for SMZ, and 6 ng L^−1^ for TYL in tap water in China, while TYL was also found in drinking water in France at 4–20 ng L^−1^ (Charuaud et al. 2019), signifying the potential for these VAs to move from farming environments to drinking water despite the application of water treatment.

In Alberta, the *Water Act* (Province of Alberta 1999) shifted the focus from supply management to the protection of aquatic and riparian ecosystems, and sustainable resource development. As such, water quality protection is the main thrust of Alberta’s irrigation strategy (Alberta Agriculture and Forestry 2020). However, surface water used for irrigation has, to date, not been analyzed for the presence of VAs. Recognizing that the SSRB represents one of the most intensively farmed agroecosystems in Canada, the objectives of this study were to measure concentrations of VAs in surface water associated with (i) beef cattle feedlot environs and (ii) irrigation conveyances, within the river basin.

## Materials and methods

### Feedlot environs

Two beef cattle feedlots with production practices typical of western Canada were selected for water sampling. Cattle were confined in open-air, earthen-floor pens arranged side-by-side with central feed alleys. In line with provincial regulations, feedlots had runoff control catch basins (CB) with storage capacities to accommodate 1 in 30-year rainfall events in 24 h, and solid manure storage and collection areas at setback distances > 30 m from common water bodies (Alberta Agriculture and Rural Development 2008).

Feedlot A (18,000 head capacity) was located in the Oldman River Basin (Fig. 1a) and previously described by Tymensen et al. (2017). Briefly, surface runoff water from two thirds of the pens drained to a large catch basin (CB-1, Fig. 1b), with the remaining pens draining to a smaller catch basin (CB-2, Fig. 1b). Accumulated water in CB-2 was periodically transferred to a primary retention pond (PRP) or to CB-1. From the PRP, water was transferred to a 2-ha constructed wetland (CW) consisting of two parallel cells populated by cattail (*Typha latifolia*). After retention in the CW, water flow was directed via a grassed waterway to a secondary retention pond (SRP). The land adjacent to the feedlot was used for corn (*Zea mays* L.) or barley (*Hordeum vulgare* L.) silage production and received annual manure applications, and intermittent irrigation with runoff water from CB-1, or water from the SRP. This land drained naturally into an ephemeral creek flowing east (Fig. 1b) at ~ 100 m south of the feedlot.

Feedlot B (15,000 head capacity) was in the Red Deer River Basin in south-central Alberta (Fig. 1a). A catch basin (CB) was situated ~ 50 m, and an ephemeral creek ~ 100 m, west of the feedlot pens (Fig. 1c). Feedlot B was surrounded by pastureland. At Feedlots A and B, water samples were collected between April and October (Table 1) each year (2014–2016), because surface water is often frozen in southern Alberta during the late fall and winter.

**Table 1:** Sampling dates and sites for veterinary antimicrobials in feedlot environs, 2014–16

Feedlot A							Feedlot B			
Site	CB-1	CB-2	PRP	CW	SRP	Creek-D		CB	Creek-U	Creek-D
2014										
Date							Date			
14 Apr	—	1	1	1	1	1	14 Apr	0^b^	1	1
28 Apr	1	1	1	1	1	1	28 Apr	0^b^	1	1
28 May	1	1	1	1	1	1	20 May	0^b^	1	1
25 Jun	1	1	1	1	1	1	11 Jun	1	0^c^	0^c^
20 Jul	1	1	1	1	1	0^c^	18 Jun	0^b^	1	1
15 Sep	1	0^b^	1	1	1	1	29 Jul	0^b^	1	1
28 Oct	1	1	1	1	1	1	11 Aug	1	0^c^	0^c^
							20 Oct	1	0^c^	0^c^
2015										
Date							Date			
27 Apr	1	1	1	2	—	1	8 Apr	0^b^	1	1
19 May	1	1	1	2	—	1	13 Apr	1	0^c^	0^c^
22 Jun	1	1	1	2	—	1	15 Apr	0^b^	1	1
27 Jul	1	1	1	2	—	0^c^	20 May	0^b^	1	1
24 Aug	1	1	1	1	—	0^c^	8 Jun	1	0^c^	0^c^
5 Oct	1	1	1	2	—	0^c^	10 Aug	1	0^c^	0^c^
							8 Sep	0^d^	1	1
							14 Sep	0^d^	1	1
							21 Sep	0^d^	1	1
							20 Oct	1	0^c^	0^c^
2016										
Date							Date			
16 Aug	1	1	1	—	—	—	19 Apr	1	0^c^	0^c^
19 Sep	1	1	1	—	—	—	3 Aug	1	0^c^	0^c^
12 Oct	1	1	1	—	—	—	29 Aug	1	0^c^	0^c^
							26 Sep	1	0^c^	0^c^
							24 Oct	1	0^c^	0^c^
Total	15	15	16	18	7	9		12	11	11

*CB*, catch basin; *PRP*, primary retention pond; *CW*, constructed wetland; *SRP*, secondary retention pond; *Creek-D*, downstream creek; *Creek-U*, upstream creek

^—^Not sampled; 0, sample unobtainable due to ^a^frozen conditions, ^b^empty catch basin, ^c^dry stream bed, and ^d^hazardous conditions, catch basin full with slippery side-slopes

### Irrigation conveyances

Surface water sampling of irrigation conveyances was part of an Irrigation Districts Water Quality (IDWQ) project which assessed the quality of irrigation water within IDs in the SSRB (Charest et al. 2015). The IDWQ project ran for 5 years (2011–2015), with salinity and concentrations of nutrients, metals, pathogens, and pesticides measured at 90 sampling sites spanning all thirteen IDs. Sampling sites fell into three conveyance categories: primary, secondary, and return (Charest et al. 2015). Primary sites were main canals where source water entered an ID, while secondary sites were canals that branched off the main canal, or were immediately downstream of a reservoir. Return sites were located at the end of an ID conveyance network, where water was no longer used for irrigation, and allowed to return to the natural drainage system. Within return sites, there were two sub-categories: (i) infrastructure returns, i.e., constructed canals, generally not influenced by surface runoff; (ii) watershed returns, i.e., natural channels that collected surface runoff from adjacent irrigated land.

**Fig. 2:**
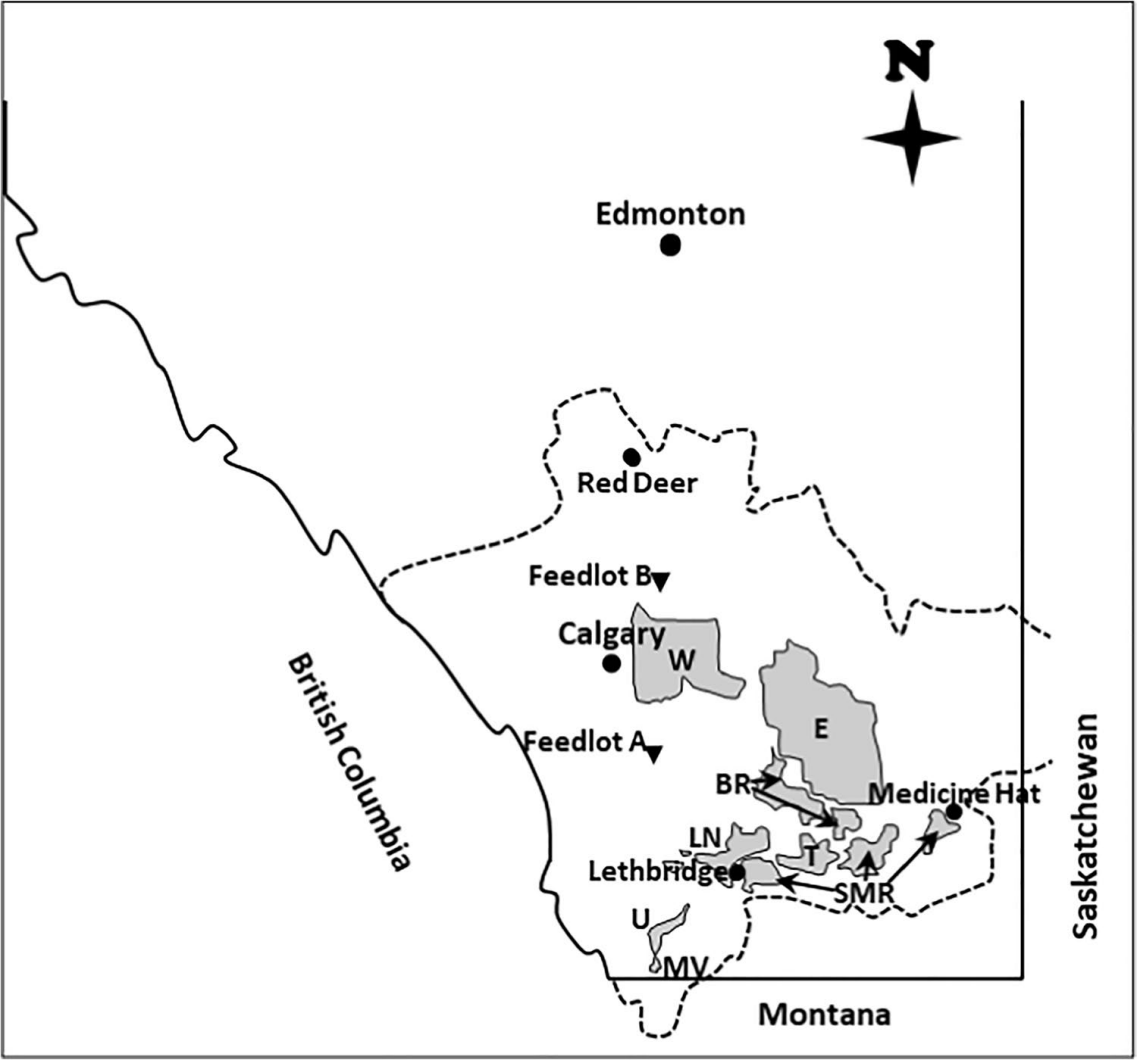
Southern Alberta showing irrigation districts (LN, Lethbridge Northern; SMR, St. Mary River; T, Taber; BR, Bow River; E, Eastern; W, Western; U, United; MV, Mountain View) sampled within the South Saskatchewan River Basin (dashed line boundary). Feedlots A and B locations included for reference

Veterinary antimicrobial analyses were added to the IDWQ project in 2013–2015 only, using subsets of sampling sites: a preliminary subset of 15 sites in 7 IDs in 2013; followed by the same 15, plus a further 9 sites (*n* = 24) in 8 IDs in both 2014 and 2015 (Table 2). The Lethbridge Northern, St. Mary River, Taber, Bow River, Eastern, and Western IDs (Fig. 2) were expected to have higher levels of VAs due to the presence of intensive livestock operations, especially Lethbridge Northern as it encompasses the highest density of beef cattle in Canada (Acharya et al. 2007). The remaining two IDs [United, and Mountain View (2014–15 only); Fig. 2] were in the less agriculturally intensive southwestern part of the SSRB (Alberta Environment 2007). These sites were envisioned as ‘controls’, with expected lower VAs concentrations due to (i) proximity to pristine headwaters from the Rocky Mountains and (ii) livestock production predominantly grazing, rather than confined feeding operations. Only secondary and return sites were chosen for VA analyses within irrigation conveyances due to the greater potential of detecting VAs because of proximity to farmland. Table 2 shows the number of sampling sites within each ID and their designations (secondary; watershed return; infrastructure return). The origin of samples by ID was 20% Lethbridge Northern; 18% St. Mary River; 13% Taber, Bow River, and Western; 12% Eastern; and 11% United-Mountain View. The origin by conveyance category was 37% secondary, 32% infrastructure return, and 31% watershed return.

**Table 2:** Number of veterinary antimicrobial sampling sites (with number of samples collected in parentheses) within the South Saskatchewan River Basin from 2013 to 2015

Irrigation district	Year	Conveyance category			Total
		Secondary	Infrastructure return	Watershed return	
Lethbridge Northern	2013	1 (2)^a^	1 (2)	1 (2)	3 (6)
	2014	2 (8)	1 (4)	2 (8)	5 (20)
	2015	2 (8)	1 (3^c^)	2 (8)	5 (19)
	Sub-total	5 (18)	3 (9)	5 (18)	13 (45)
St. Mary River	2013	– ^b^	2 (3^d^)	2 (4)	4 (7)
	2014	– ^b^	2 (8)	2 (8)	4 (16)
	2015	– ^b^	2 (8)	2 (8)	4 (16)
	Sub-total	– ^b^	6 (19)	6 (20)	12 (39)
Taber	2013	2 (4)	– ^b^	– ^b^	2 (4)
	2014	2 (8)	1 (4)	– ^b^	3 (12)
	2015	2 (8)	1 (4)	– ^b^	3 (12)
	Sub-total	6 (20)	2 (8)	– ^b^	8 (28)
Bow River	2013	1 (2)	– ^b^	1 (2)	2 (4)
	2014	2 (8)	– ^b^	1 (4)	3 (12)
	2015	2 (8)	– ^b^	1 (4)	3 (12)
	Sub-total	5 (18)	– ^b^	3 (10)	8 (28)
Eastern	2013	– ^b^	– ^b^	1 (2)	1 (2)
	2014	1 (4)	1 (4)	1 (4)	3 (12)
	2015	1 (4)	1 (4)	1 (4)	3 (12)
	Sub-total	2 (8)	2 (8)	3 (10)	7 (26)
Western	2013	1 (2)	– ^b^	1 (2)	2 (4)
	2014	2 (8)	– ^b^	1 (4)	3 (12)
	2015	2 (8)	– ^b^	1 (4)	3 (12)
	Sub-total	5 (18)	– ^b^	3 (10)	8 (28)
United	2013	– ^b^	1 (2)	– ^b^	1 (2)
	2014	– ^b^	2 (8)	– ^b^	2 (8)
	2015	– ^b^	2 (8)	– ^b^	2 (8)
	Sub-total	– ^b^	5 (18)	– ^b^	5 (18)
Mountain View	2013	– ^b^	– ^b^	– ^b^	– ^b^
	2014	– ^b^	1 (4)	– ^b^	1 (4)
	2015	– ^b^	1 (3^e^)	– ^b^	1 (3)
	Sub-total	– ^b^	2 (7)	– ^b^	2 (7)
Grand total		23 (82)	20 (69)	20 (68)	63 (219)

^a^Values in parentheses represent number of water samples collected based on two samplings site^−1^ (June, August) in 2013, and four samplings site^−1^ (June, July, August, September) in 2014 and 2015

^b^‘– ‘ no sample collected. One site not sampled: ^c^June 2015, ^d^June 2013, and ^e^September 2015

### Water sample collection

Over the 3 years (2014–2016), 80 surface water samples were collected from Feedlot A, and 34 from Feedlot B, resulting in a total of 114 samples (Table 1). In 2014, there were six sampling sites at Feedlot A: CB-1, CB-2, PRP, CW, SRP, and the ephemeral creek, 2.6 km downstream (Creek-D) from the feedlot (Fig. 1b, Table 1). For the CW, four locations per cell were collected and composited into one sample. In 2015, two samples were collected from the CW, one from each cell, except on 24 August, when only one cell was sampled (Table 1). The SRP site was dropped from the study in 2015 (Table 1) due to potential variation in water quality because the water flows into the SRP not only originated from the CW, but also intermittently from the PRP via overland flow. In 2014 and 2015, not all sites were sampled on each date (Table 1) at Feedlot A, due to frozen or dry catch basins, or a dry ephemeral creek. In 2016, sampling at Feedlot A was confined to only three sites (CB-1, CB-2, PRP), later in the season (Table 1).

Feedlot B had three sampling sites: the catch basin (CB); and sites on the ephemeral creek located ~ 1.6 km upstream (Creek-U), and ~ 1.5 km downstream (Creek-D), from the feedlot (Fig. 1c, Table 1). Due to dry conditions, samples could not be collected from the CB on some dates in 2014 and 2015 or from the ephemeral creek in all 3 years.

For irrigation conveyances, water sampling campaigns extended to 3–4 d to complete all 90 sites of the larger IDWQ project. Samples were collected twice in 2013 (11–13 June; 27–29 August), four times in 2014 (10–12 June; 7–10 July; 5–7 August; 2–4 September) and four times in 2015 (8–11 June; 6–9 July; 27–30 July, 31 August–September 3) (Table 2). For year-to-year comparisons, sampling times were designated by month. Since two samplings occurred in July 2015, the latter one (27–30 July) was assigned to August.

All water samples were collected at mid-depth using 1-L polyethylene bottles attached to a telescopic pole. At feedlots, water from four locations per sampling site was deposited into a clean plastic pail, from which 1 L was funneled into pre-cleaned amber glass bottles, suitable for trace organic compound analysis. At irrigation conveyances, 1-L samples were collected mid-channel and transferred into 1-L amber glass bottles. In the field, water samples were placed in iced coolers and then refrigerated at 4 ℃ prior to transport to the National Hydrology Research Centre, Environment and Climate Change Canada, Saskatoon, SK. Samples were extracted for VAs within 24–48 h after arrival at the laboratory.

### Sample analyses for veterinary antimicrobials

Six VAs were quantified in irrigation conveyance water samples in 2013: CTC, SMZ, TYL, MON, LIN, and ERY. Analysis of a seventh VA, tetracycline (TC), was added for all 2014 and 2015 samples (feedlot and irrigation conveyance), and 2016 feedlot samples. Among the seven analyzed, four VAs were administered to cattle at Feedlots A and B (CTC, SMZ, TYL, MON), while three were not (LIN, ERY, TC). Water samples were subjected to solid phase extraction (SPE) followed by liquid chromatography-tandem mass spectrometry (LC–MS-MS) for detection and quantification of VA concentrations.

### Solid-phase extraction

Water samples (500 mL) were transferred to graduated cylinders and mixed thoroughly with 25 mL of 0.2 M citric acid (pH 4.7) buffer (2013 samples), or McIlvaine-EDTA (50 mL L^−1^ sample) buffer (2014–15, 16 samples). McIlvaine-EDTA buffer (pH 4.0) was prepared by dissolving anhydrous dibasic sodium phosphate (28.4 g) in distilled water (1 L) to create a phosphate solution. Citric acid monohydrate (21 g) was dissolved in distilled water (separate 1 L) to which phosphate solution (625 mL) was added and mixed thoroughly. Disodium EDTA (ethylene-diamine-tetra-acetic acid) dehydrate (60.5 g) was then added to the resulting 1.625 L solution.

Solid-phase extraction of the buffered samples was carried out using conditions modified from Cessna et al. (2011) and Jacobsen et al. (2004). For 2013 samples, SPE was conducted using an Oasis weak cation exchange (WCX) cartridge (60-µm particle size, 225 mg sorbent, Waters, Milford, MA) stacked on top of an Oasis hydrophilic-lipophilic balance (HLB) cartridge (60-µm particle size, 225 mg sorbent, Waters, Milford, MA). For the 2014–2016 samples, Strata strong anion exchange (SAX) cartridge (55-µm particle size, 500 mg sorbent, Phenomenex, Torrance, CA) was used instead of WCX. The SAX cartridge was stacked on top of an Oasis hydrophilic-lipophilic balance (HLB) cartridge. Both cartridges were pre-conditioned in tandem with methanol (10 mL) followed by de-ionized water (10 mL). Buffered water samples were then passed through the cartridges under vacuum at a rate of 100 mL h^−1^, followed by de-ionized water (10 mL) to remove salts. Cartridges were air-dried for 30 s under vacuum and maintained at − 10℃ until elution.

The cartridges were separated and eluted within 24 h. The WCX/SAX cartridge was eluted with methanol (5 mL) followed by methanol containing 2% formic acid (5 mL) whereas the HLB cartridge was eluted only with methanol (10 mL). Eluents from the WCX or SAX and HLB cartridges were collected separately into 15 mL centrifuge tubes and concentrated to ~ 500 µL under a steady stream of N_2_ gas (water bath at 40 ℃). The extract residues were combined, taken to 1 mL with de-ionized water, vortexed, and transferred into a 2-mL LC vial through a 0.45-µm nylon membrane syringe filter (Chromatographic Specialties Inc., Brockville, Ontario, Canada) equipped with a 3-mL disposable syringe (BD Diagnostics, Mississauga, Ontario, Canada). The combined extract residues were fortified with 10 µL of 10 mg L^−1 13^C_6_-sulfamethazine (internal standard; Cambridge Isotope Laboratories, Andover, MA) prior to analysis to normalize for variation in ionization within the ion source of the mass spectrometer. Calibration curves for each VA were created using ratios of peak areas of analyte and internal standard (analyte signal/internal standard signal). Similarly, the concentration of the VA in an unknown sample was calculated using ratios of peak areas of analyte and internal standard.

### Liquid chromatography-tandem mass spectrometry (LC–MS-MS) analysis

All concentrated extracts were analyzed using a high-pressure liquid chromatograph (Waters 2965 Alliance Separation Module, Waters Canada, Mississauga, ON) interfaced with a tandem mass spectrometer (Micromass Quattro Ultima, Waters Canada, Mississauga, ON). The conditions for LC–MS-MS analysis were adapted from Cessna et al. (2011). Liquid chromatographic separation of analytes was achieved using a 50-mm × 2.1-mm i.d. stainless steel column (Kinetex biphenyl, 2.6-µm diameter particle packing, Phenomenex, Torrance, CA), a mobile phase flow rate of 0.2 mL min^−1^, and an injection volume of 20 µL. Two mobile phases were used: mobile phase A was 100% de-ionized water containing 0.1% formic acid (v/v), and mobile phase B was 90% acetonitrile [10% de-ionized water containing 0.1% formic acid (v/v)]. Gradient elution (Table S1) was used to achieve separation of analytes prior to detection by tandem mass spectrometry. Retention times of all analytes are listed in Table S3.

Mass spectrometer parameters were optimized by infusion of individual standard analyte solutions. Individual stock solutions of analytical standards were prepared in acetonitrile (100 mg L^−1^). A working solution mixture of all analytes was made from the stock solutions in de-ionized water (1 mg L^−1^), and calibration standards were prepared. The linearity of the instrument and method working range was established using a six-point calibration curve (2.5, 5, 10, 25, 50, and 100 ng mL^−1^, *r*^2^ > 0.92) for each analyte. Repeatability of the method was assessed using 6 calibration standards (2.5 ng mL^−1^, 5, 10, 25, 50, and 100 ng mL^−1^) which were analyzed five times over a period of 5 days (*n* = 5 for each concentration level) and percent relative standard deviations (RSD %) were calculated (Table S2). The stability of the retention times of all analytes was also assessed (calculated RSD 0.1 to 0.9%) using the abovementioned repeated calibration standard analysis. Calibration standards and sample extracts were analyzed simultaneously where every ten sample extracts were sandwiched between two sets of calibration standards and the linearity of the calibration standards curve was assessed (*r*^2^ > 0.92).

Water samples were analyzed in sets of 8 along with 2 fortified and 2 control samples. Control water samples (from Swift Current Creek, Swift Current, SK, Canada) were fortified with 10 or 50 µL of an aqueous solution of a mixture of iso-chlortetracycline, sulfamethazine, tylosin, monensin, lincomycin, erythromycin, or tetracycline, each at 1 mg L^−1^ (equivalent to 10 or 100 ng L^−1^). The fortified water was thoroughly mixed and subjected to SPE under the same conditions as described earlier. Control samples served as sample blanks comparable to sample matrix without analytes and extracted and analyzed similarly to water samples from the study. Control samples did not contain any traces of the seven VAs. The extraction method performance was evaluated with analyte recovery values, which ranged from 46 to 105%, when fortified at 10 ng L^−1^ and from 48 to 110%, when fortified at 100 ng L^−1^, linearity of calibration curves, LOQ, and method detection limit (MDL). Solvent blanks were employed at regular intervals, throughout the instrument analysis process to account for analyte carryover. The LOQ was determined based on the lowest analyte concentration which yielded a well-resolved chromatographic peak with a signal-to-noise ratio of 10 and reproducible with ± 20% whereas MDL was determined as the lowest detectable concentration using a blank water sample processed similar to samples. The limits of quantification (LOQ) for each analyte are shown in Table S3. The extraction method efficiency using the SAX cartridge was similar to that with WCX, for all analytes except for CTC at 100 ng L^−1^. The recoveries for CTC at 100 ng L^−1^ were 58 ± 6 (WCX) and 66 ± 8 (SAX). The reported analyte concentrations were not normalized to respective analyte recoveries.

Precursor and product ion transitions used for confirmation and quantification are listed in Table S3. The sum of two product ion transitions for each analyte was used for quantification and data analysis was carried out using MassLynx software (v 4.1, Waters, Milford, MA).

Detection frequencies (> LOQ) of seven antimicrobials in water samples from feedlot sampling sites, from 2014 to 2016, are listed in Table S4.

### Precipitation data

For feedlots, daily precipitation data were obtained from weather stations (Alberta Climate Information Service 2020) located closest to Feedlot A (14 km) and Feedlot B (12.8 km). For IDs, one centrally located weather station in each of the eight IDs was chosen and mean precipitation was estimated.

### Statistical analyses

Concentrations of VAs falling between 50 and 100% of LOQ (2.5–5 ng L^−1^) were assigned values equivalent to the MDL (method detection limit, 2.5 ng L^−1^), and were included in statistical analyses performed by SigmaPlot (Systat Software Inc. 2020). Concentrations < MDL were considered undetectable and excluded from statistical analyses.

For statistical comparison, concentrations of individual VAs in feedlot samples were pooled by year (2014–2016; *n* = 3), by sampling month (April–October, *n* = 7), and by sample source [catch basin (CB1, CB2, Feedlot A; CB, Feedlot B), retention pond/wetland (PRP, CW, and SRP, Feedlot A), and creek (Creek-U, Feedlot A; Creek-U and Creek D, Feedlot B), *n* = 3]. Individual VA concentration data for irrigation conveyances were pooled for statistical comparison of sampling time [*n* = 10, (8 for TC)], ID (*n* = 7; data from the United and Mountain View IDs amalgamated as a control), and conveyance category/sub-categories (secondary site, infrastructure return, watershed return; *n* = 3). Data were compared using side-by-side box plots which generated descriptive statistics as well as visual interpretation. The Kruskal–Wallis test was used to identify significant differences between median VA concentrations. A non-parametric one-way ANOVA was selected because VAs concentrations were not always normally distributed. Following a significant Kruskal–Wallis test (*p* < 0.05), pairwise multiple comparison analyses were performed with a post-hoc Dunn’s test. Median concentrations of VAs were compared between upstream and downstream creek locations at Feedlot B using the Mann–Whitney rank sum test. The Mann–Whitney rank sum test was also used to compare median VA concentrations of feedlot environ samples to irrigation conveyance samples.

## Results and discussion

### Feedlot environs

Overall, detection frequency in feedlot water samples was 100% for CTC and TC, followed by 94% for MON, 84% for TYL, 72% for LIN, 66% for ERY, and 23% for SMZ (Table 3). Sulfamethazine showed the highest proportions of samples with MDL–LOQ detections (11%) or undetectable (66%). The proportion of samples undetectable for LIN and ERY ranged from 23–26% (Table 3). Maximum concentrations (Table 4) ranked from 1384 μg L^−1^ for TC (PRP, Feedlot A; 27 July 2015), to 215 ng L^−1^ for MON (SRP, Feedlot A; 28 October 2014), 4,861 ng L^−1^ for CTC (CB-1, Feedlot A; 5 October 2015), 951 ng L^−1^ for TYL (SRP, Feedlot A; 28 October 2014), 166 ng L^−1^ for ERY (CW, Feedlot A; 15 September 2014), 117 ng L^−1^ for LIN (Creek-U, Feedlot B; 14 April 2014), and 17 ng L^−1^ for SMZ (CB-2, Feedlot A; 24 August 2015)]. Median concentrations followed a similar order, ranging from 531 ng L^−1^ for TC to 6 ng L^−1^ for SMZ (Table 4). Minimum concentrations were < LOQ for four VAs (SMZ, TYL, LIN, ERY), while CTC (Creek-D, Feedlot A; 28 April 2014) and MON (Creek-D, Feedlot B; 8 April 2015) both had minimum concentrations of 5.6 ng L^−1^ (Table 4). The minimum concentration observed for TC was 13 ng L^−1^ (Creek-D, Feedlot B; 28 April 2014).

**Table 3:** Detection frequency parameters of seven antimicrobials in water samples from feedlot environs, 2014 to 2016

Sample group	Samples, *n*	CTC	SMZ	TYL	MON	LIN	ERY	TC	Overall mean^a^	No. VAs detected
		Undetectable frequency < MDL (2.5 ng L^−1^) [%]								
All	114	0	66	8	6	23	26	0	18	
		Detection frequency MDL–LOQ (2.5–5 ng L^−1^) [%]								
All	114	0	11	8	0	5	8	0	5	
		Detection frequency > LOQ (5 ng L^−1^) [%]								
All	114	100	23	84	94	72	66	100	77	7
Year										
2014	52	100	33	81	94	87	71	100	81	7
2015	51	100	16	84	92	61	53	100	72	7
2016	11	100	9	100	100	55	100	100	81	7
Month										
April	27	100	15	85	85	48	22	100	65	7
May	16	100	13	87	100	81	88	100	81	7
June	18	100	0	83	100	83	100	100	81	6
July	10	100	70	90	100	100	90	100	93	7
August	11	100	27	100	100	73	55	100	79	7
September	15	100	20	60	80	53	60	100	68	7
October	17	100	41	88	100	88	77	100	85	7
Source										
Catch basin^b^	42	100	31	98	100	76	81	100	84	7
Retention pond/wetland^c^	41	100	32	93	100	85	71	100	83	7
Creek^d^	31	100	0	55	77	48	39	100	60	6

^a^Based on 7 analyses (CTC, SMZ, TYL, MON, LIN, ERY, TC) of 114 samples (*n* = 7 × 114 = 798)

^b^Includes Catch basins 1 and 2 (CB-1, CB2), Feedlot A; and catch basin (CB), Feedlot B

^c^Includes primary retention pond (PRP), constructed wetland (CW), and secondary retention pond (SRP) at Feedlot A

^d^Includes Creek-downstream (Creek-D), Feedlot A; and Creek-downstream (Creek-D) and upstream (Creek-U) at Feedlot B

**Table 4:** Summary statistics for concentrations of seven antimicrobials in feedlot environs and irrigation conveyances

Statistic	CTC	SMZ	TYL	MON	LIN	ERY	TC
Feedlot environs^a^ (ng L^−1^)							
Maximum M	4861	17	951	5215	117	166	1,384,822
Median	277	6.0	42	436	14	9.9	531
Mean	479	6.9	114	801	19	23	50,010
Minimum	5.6	< LOQ	< LOQ	5.6	< LOQ	< LOQ	13
10th percentile	35	< LOQ	5.2	14	5.4	< LOQ	92
25th percentile	113	< LOQ	10	116	8.1	6.8	300
75th percentile	537	9.6	132	938	27	25	1,950
90th percentile	1001	13	324	2842	39	49	11,045
Irrigation conveyances^b^ (ng L^−1^)							
Maximum M	69	33	117	31	< LOQ	29	155
Median	25	5.5	< LOQ	< LOQ	< LOQ	5.6	56
Mean	27	9.7	15	4.8	< LOQ	6.7	59
Minimum	< LOQ	< LOQ	< LOQ	< LOQ	< LOQ	< LOQ	19
10th percentile	10	< LOQ	< LOQ	< LOQ	< LOQ	< LOQ	28
25th percentile	17	< LOQ	< LOQ	< LOQ	< LOQ	< LOQ	37
75th percentile	35	17	7.9	6.1	< LOQ	9.9	76
90th percentile	47	30	70	8.9	< LOQ	13	92

^a^Based on 114 water samples

^b^Based on 219 water samples (190 for TC)

**Fig. 3:**
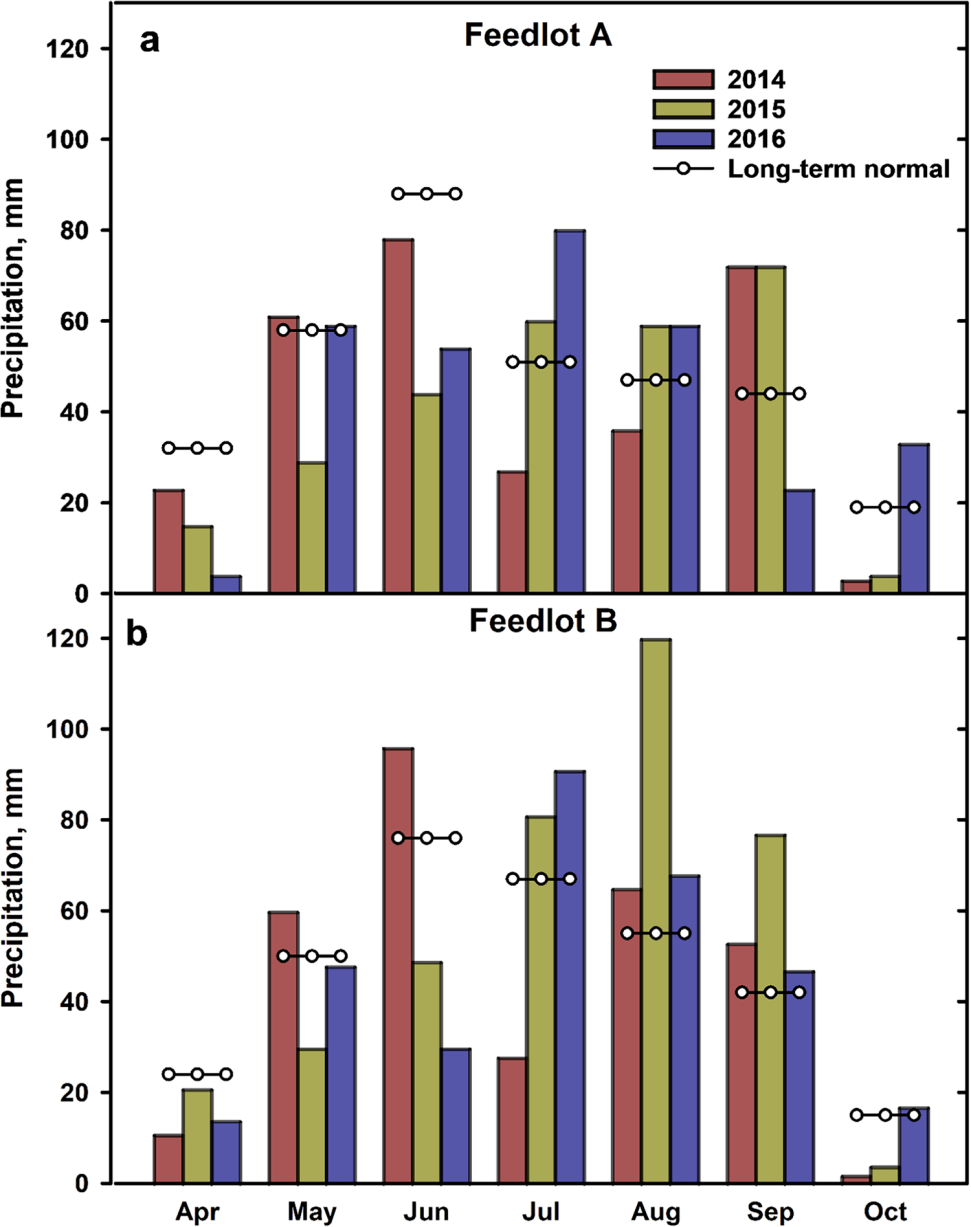
Monthly precipitation (April − October, 2014, 2015, 2016; long-term normal) at **a** Feedlot A and **b** Feedlot B. Source: Alberta Climate Information Service stations located 14 km from Feedlot A and 12.8 km from Feedlot B (http://agriculture.alberta.ca/acis/weather-data-viewer.jsp)

**Fig. 4:**
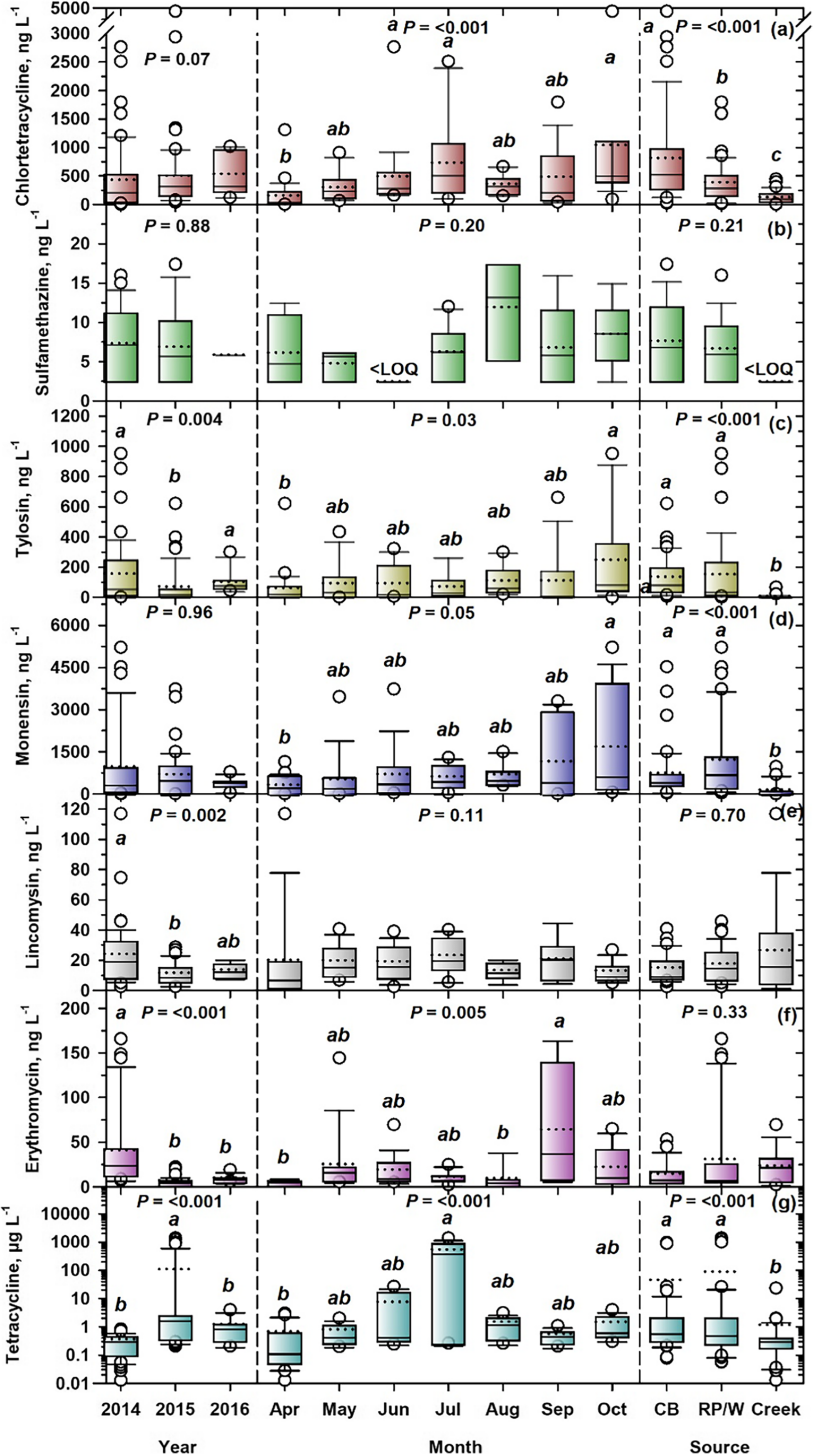
Box plots for concentrations of veterinary antimicrobials in feedlot environs: **a** CTC, **b** SMZ, **c** TYL, **d** MON, **e** LIN, **f** ERY, and **g** TC, grouped by sampling year, sampling month, and sample source (CB, catch basins at Feedlots A and B; RP/W, retention pond/wetland: PRP, CW, and SRP at feedlot A; Creek, Creek-D at feedlot A, Creek-U and Creek-D at feedlot B). Within boxes, a solid horizontal line marks the median concentration. A dotted horizontal line marks the mean concentration. The lower boundary of the box indicates the 25th percentile and the upper boundary the 75th percentile. Whiskers (error bars) indicate 10th and 90th percentiles. Circle symbols indicate outliers falling outside the 10th and 90th percentiles. *P* values are presented for Kruskal–Wallis tests on each group of samples. When significant (*P* < 0.05), box plots within groups with different letters have significantly different median concentrations based on Dunn’s test

Since CTC and TC were both detected at 100%, there was no temporal or spatial variation in their detection frequencies (Table 3). Comparing 2014 and 2015 (years with similar numbers of samples (*n* = 51–52)), SMZ detection frequency was higher in 2014 (33%) than 2015 (16%), as were LIN (87 vs. 61%), and ERY (71 vs. 53%) (Table 3). Sampling year was significant for median concentrations of TYL (Fig. 4c), LIN (Fig. 4e), ERY (Fig. 4f), and TC (Fig. 4g), but not CTC (Fig. 4a), SMZ (Fig. 4b), or MON (Fig. 4d). For TYL (Fig. 4c), both 2014 and 2016 had significantly higher median concentrations (61–85 ng L^−1^) than 2015 (23 ng L^−1^). 2014 was also significantly higher for LIN (*x̃* = 20 ng L^−1^) than 2015 (10 ng L^−1^) (Fig. 4e), as was ERY (26 vs. 7 ng L^−1^) (Fig. 4f). At Feedlot A, May–June precipitation in 2014 totaled 139 mm, compared to 73 mm in 2015 (Fig. 3a). At Feedlot B, precipitation in May–June 2014 (Fig. 3b) was double (156 mm) that of 2015 (78 mm). This may explain the higher detection frequencies of SMZ, LIN, and ERY (*x̄* = 64%) in 2014, than 2015 (*x̄* = 43%), and significantly higher median concentrations of TYL, LIN, and ERY in 2014 (*x̄* = 36 ng L^−1^), than 2015 (*x̄* = 13 ng L^−1^). Higher precipitation likely led to greater surface runoff from (i) feedlot pen floors and bedding packs, which are collected in catch basins (Miller et al. 2004) and (ii) manured land which contributed to VAs in creek settings. Little et al. (2007) found that the majority of surface runoff in Alberta occurred in spring, coinciding with land application of fresh manure, just prior to seeding, when soil is not frozen or snow-covered. TC was different in that 2015 had a significantly higher median concentration (1.96 μg L^−1^) than both 2014 and 2016 (0.36–0.98 μg L^−1^) (Fig. 4g).

Monthly variation in detection frequency was most evident for SMZ, varying from 0% in June to 70% in July (Table 3). April detections were lowest for both LIN (48%) and ERY (22%), before increasing to 100% for ERY in June, and for LIN in July, and then falling to 55% for ERY in August, and 53% for LIN in September (Table 3). Overall, April showed the lowest detection frequency (65%), while frequencies increased from May–June (81%) through July (93%) (Table 3). The lower detection frequency in April (65%) was matched by significantly lower concentrations of CTC, TYL, MON, ERY, and TC in April than in other months. For example, April was significantly lower than June, July, and October for CTC (46 vs. 290–513 ng L^−1^; Fig. 4a); October for both TYL (28 vs. 89 ng L^−1^; Fig. 4c) and MON (260 vs. 656 ng L^−1^; Fig. 4d); September for ERY (7 vs. 39 ng L^−1^; Fig. 4f); and July for TC (0.13 vs. 447 μg L^−1^; Fig. 4g). This was attributed to precipitation patterns (Fig. 3) and associated runoff, with mean April precipitation 76% lower (*x̄* = 14 mm; 2 feedlots × 3 years, *n* = 6) than May–September (*x̄* = 59 mm; 5 mo × 2 feedlots × 3 years, *n* = 30). These findings agreed with Hyland et al. (2003), who showed that fecal contamination in surface waters from the Oldman River Basin was lower during winter than in summer months (May–September). In an intensively agricultural watershed in Nebraska, the highest mean concentrations of MON (49 ng L^−1^) and LIN (68 ng L^−1^) also occurred in in summer months (Jaimes-Correa et al. 2015). The sampling month was non-significant for median concentrations of SMZ (2.5–12 ng L^−1^; Fig. 4b), and LIN (8–22 ng L^−1^; Fig. 4e).

Detection frequencies were similar for samples sourced from catch basin and retention ponds/wetland for SMZ (31–32%), TYL (93–98%), and MON (100%), while creeks were zero for SMZ, or substantially lower for TYL (55%) and MON (77%) (Table 3). Creeks also showed lower detection frequencies of both LIN and ERY (39–48%), compared to catch basins or retention ponds/wetlands (71–85%). The lower detection frequencies in creeks than in feedlot catch basins or retention ponds/wetlands were expected, because runoff carrying VAs from feedlot pens should not be entering creeks, if catch basins are performing optimally, and best management practices are followed.

Four VAs showed significant effects of feedlot sample source on median concentrations: CTC (Fig. 4a), TYL (Fig. 4c), MON (Fig. 4d), and TC (Fig. 4g). Only CTC showed a significant downward trend from catch basin (525 ng L^−1^) to retention ponds/wetlands (295 ng L^−1^) and to creeks (99 ng L^−1^) (Fig. 4a). The 44% reduction in the median concentration of CTC in retention ponds/wetland samples, compared to catch basin samples, showed that the CW played a role in mitigating CTC levels. In addition, Tymensen et al. (2017) isolated significantly fewer enterococci and *E. coli* from the CW compared to catch basin B, which was the input source for the wetland. Constructed wetlands are globally recognized as a treatment technology for many types of wastewater (Vymazal 2011; Ilyas et al. 2020). The elimination of VAs from CWs can be achieved through physicochemical processes including absorption, biotransformation, decomposition, photodegradation, adsorption by wetland soil and plants, and microbial biodegradation (Choi et al. 2016; Hsieh et al. 2015; Kadlec 1992), with removal efficiencies of 20–50% (Almeida et al. 2013; Li et al. 2014). Environmental factors including presence of nitrate and humic substances affect the photodegradation of some VAs (Sun et al. 2014). In addition, phytoremediation of VAs from animal liquid waste has shown to be promising (Hu et al. 2020). Cessna et al. (2020) reported DT_50_ (time required for 50% dissipation) values of 3.3 d for CTC, 7 d for SMZ, and 14 d for LIN for Canadian prairie wetlands. However, in our study, apart from CTC, none of the other VAs (SMZ, TYL, MON, LIN, ERY, TC) showed evidence of removal in the CW, with no significant difference between concentrations in catch basin vs. retention ponds/wetland samples. Further research is required to evaluate the dissipation of VAs in CW and catch basins. For three VAs, creek water concentrations were significantly lower than both catch basin and retention ponds/wetland: 7 vs. 43–88 ng L^−1^ for TYL (Fig. 4c), 19 vs. 456–731 ng L^−1^ for MON (Fig. 4d), and 0.36 vs. 0.59–0.69 μg L^−1^ for TC (Fig. 4g). Concentrations of the remaining VAs were not significantly affected by feedlot sample source, with relatively low median values falling within narrow ranges: SMZ, 2.5–7 ng L^−1^ (Fig. 4b); LIN, 10–17 ng L^−1^ (Fig. 4e), and ERY, 9–24 ng L^−1^ (Fig. 4f). Notably LIN and ERY were not administered to cattle in either feedlot, which suggests low concentrations of these VAs in feedlot water samples (catch basins, retention ponds/wetland, adjacent creeks) were due to other sources in the catchments, possibly from swine operations.

For the creek at Feedlot B, higher downstream vs. upstream detection frequency for TYL (73 vs. 36%) and MON (82 vs. 64%) [Table S4], did not lead to significantly higher downstream concentrations of these VAs. In fact, none of six detected VAs showed a creek location effect (*P* = 0.15–0.83) on median concentration, which was unanticipated, as degradation of water quality (e.g., increased nutrient and pathogen loads), linked to intensive livestock production, has been widely reported within the SSRB (Byrne et al. 2006; Johnson et al. 2003; Jokinen et al. 2012). Our results point to possible entry of VAs further upstream than our sampling location, or a limited contribution of Feedlot B to overall VAs loads in the adjacent creek.

### Irrigation conveyances

For irrigation conveyance samples, overall detection frequencies ranked from ubiquitous (100%) for TC to 94% for CTC, with substantially lower detection frequencies for ERY (18%), TYL (15%), MON (10%), and SMZ (4%) (Table 5). Lincomycin was undetectable (< LOQ) in irrigation conveyance water. Substantial proportions of conveyance samples analyzed for TYL (26%) and MON (21%) fell into the MDL–LOQ detection category (used in statistical analyses of concentration data).

**Table 5:** Detection frequency of seven antimicrobials in water samples from irrigation conveyances, 2013 to 2015

Sample group	Samples, *n*^a^	CTC	SMZ	TYL	MON	LIN	ERY	TC	Overall mean^b^	No. VAs detected
		Undetectable frequency < MDL (2.5 ng L^−1^) [%]								
All	219 (190)	1	94	59	69	93	69	0	56	
		Detection frequency MDL–LOQ (2.5–5 ng L^−1^) [%]								
All	219 (190)	5	2	26	21	7	13	0	11	
		Detection frequency > LOQ (5 ng L^−1^) [%]								
All	219 (190)	94	4	15	10	0	18	100	33	6
Sampling time										
Jun 2013	14	93	0	93	14	0	71	–^c^	45	4
Aug 2013	15	40	27	100	0	0	100	–^c^	44	4
Jun 2014	24	100	0	0	0	0	13	100	30	3
Jul 2014	24	100	0	0	0	0	8	100	30	3
Aug 2014	24	100	0	0	13	0	0	100	30	3
Sep 2014	24	100	0	4	8	0	38	100	36	5
Jun 2015	23	100	0	4	22	0	0	100	32	4
Jul 2015	24	92	13	4	13	0	0	100	32	4
Aug 2015	24	100	0	0	13	0	0	100	30	3
Sep 2015	23	100	4	13	22	0	0	100	34	5
Irrigation district										
Lethbridge Northern	45 (39)	91	2	13	2	0	27	100	32	6
St. Mary River	39 (32)	92	0	23	8	0	21	100	33	5
Taber	28 (24)	96	7	18	21	0	18	100	36	6
Bow River	28 (24)	96	4	14	25	0	18	100	35	6
Eastern	26 (24)	96	12	12	4	0	15	100	33	6
Western	28 (24)	96	4	18	11	0	7	100	32	6
United-Mountain View	25 (23)	96	0	8	8	0	12	100	31	5
Conveyance category										
Secondary	82 (72)	96	6	15	11	0	15	100	34	6
Infrastructure return	69 (62)	94	3	12	12	0	17	100	33	6
Watershed return	68 (56)	93	1	21	9	0	22	100	33	6

^a^TC in parentheses

^b^Based on 6 analyses (CTC, SMZ, TYL, MON, LIN, ERY) of 219 samples and 1 analysis (TC) of 190 samples [*n* = (6 × 219) + (1 × 190) = 1504]

^c^Not analyzed

Rankings for maximum concentrations were of the order: 155 ng L^−1^ for TC (secondary site, Bow River ID, September 2014), 117 ng L^−1^ for TYL (secondary site, Western ID, September 2014), 69 ng L^−1^ for CTC (watershed return, Bow River ID, September 2014), 33 ng L^−1^ for SMZ (infrastructure return, Eastern ID, July 2015), 31 ng L^−1^ for MON (watershed return, Western ID, June 2013), and 29 ng L^−1^ for ERY (secondary site, Taber ID, June 2013) (Table 4). It was noteworthy that maximum concentrations of three VAs (CTC, TYL, TC) occurred in the September 2014 sampling and two VAs (MON, ERY) in the June 2013 sampling. The 21-day precipitation in the SSRB prior to and including sampling days (Table S5) was 78–79 mm for the September 2014 and June 2013 samplings, substantially higher than all other samplings (3–34 mm).

**Fig. 5:**
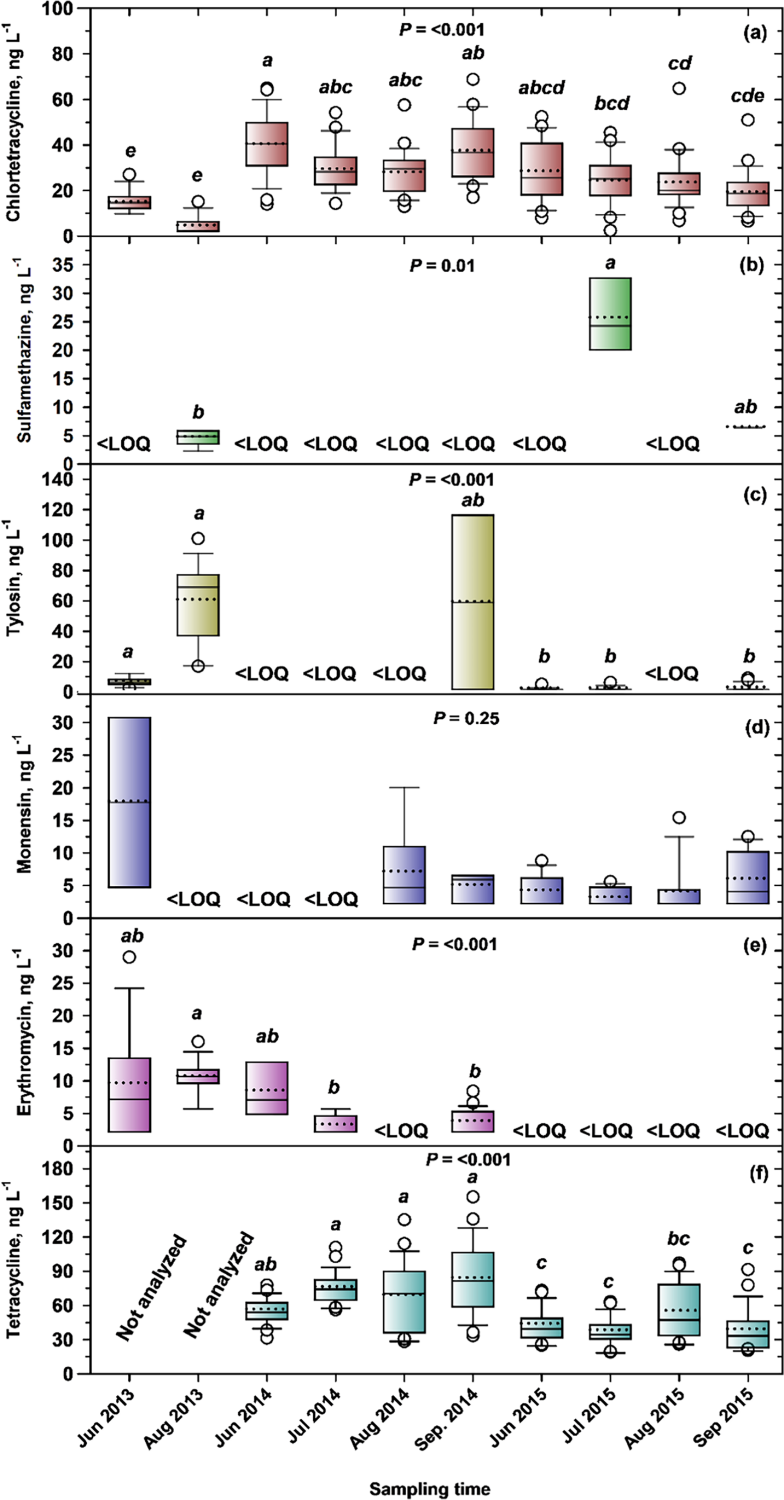
Box plots for concentrations of veterinary antimicrobials in irrigation conveyances: **a** CTC, **b** SMZ, **c** TYL, **d** MON, **e** ERY, and **f** TC, grouped by sampling time. Within boxes, a solid horizontal line marks the median concentration. A dotted horizontal line marks the mean concentration. The lower boundary of the box indicates the 25th percentile and the upper boundary the 75th percentile. Whiskers (error bars) indicate 10th and 90th percentiles. Circle symbols indicate outliers falling outside the 10th and 90th percentiles. *P* values are presented for Kruskal–Wallis tests. When significant (*P* < 0.05), box plots with different letters have significantly different median concentrations based on Dunn’s test

Sampling time had a significant effect on median concentrations of CTC (Fig. 5a), SMZ (Fig. 5b), TYL (Fig. 5c), ERY (Fig. 5e), and TC (Fig. 5f), but not MON (Fig. 5d). For CTC, both 2013 samplings were significantly lower (*x̃* = 2.5–15 ng L^−1^) than all other samplings (20–41 ng L^−1^), except September 2015 (19 ng L^−1^) (Fig. 5a). Comparing median concentrations in equivalent months, September 2014 was significantly higher than September 2015, for both CTC (37 vs. 19 ng L^−1^, Fig. 5a) and TC (84 vs. 36 ng L^−1^, Fig. 5f). This was likely explained by higher 21-day precipitation prior to September 2014 (78 mm) than September 2015 (29 mm) samplings [Table S5]. Also for TC (Fig. 5f), 80% higher 1 May–31 July precipitation (Table S5) in 2014 (196 mm), than 2015 (109 mm), in the SSRB, likely explained a 60% higher mean median concentration for June–August 2014 (69 ng L^−1^) vs. June–August 2015 (43 ng L^−1^). These findings agreed with other studies where higher VA concentrations were recorded under high-flow conditions associated with high seasonal rainfall and surface runoff (Forrest et al. 2011; Jaimes-Correa et al. 2015). In southern Alberta, Gannon et al. (2005) suggested aggregation and accumulation of particulate bacteria in sediments that accumulated in areas of low water flow, e.g., slow-moving stretches of rivers, and behind weirs and dams in irrigation conveyances. However, during spring runoff and following summer storm events, increased flow rates and scouring within the drainage system contributed to increased bacterial contamination of surface water. The same mechanism may be true for VAs.

However, not all effects on VA detection frequencies and concentrations were explained by precipitation and its influence on surface runoff. For example, the August 2013 sampling had the lowest 21-day precipitation (3 mm, Table S5), yet produced 100% detection frequencies, and significantly higher median concentrations of TYL (70 ng L^−1^) than June, July, and September 2015 (*x̃* = 2.5 ng L^−1^) (Fig. 5c), and ERY (*x̃* = 11 ng L^−1^) than July and September 2014 (*x̃* = 2.5 ng L^−1^) (Fig. 5e), as well as the highest detection frequency (27%) of SMZ (Table 5). This agreed with Alonso et al. (2019) who found higher MON concentrations with low rainfall conditions. Conversely, with high rainfall, they reported greater runoff and higher discharge in rivers, which increased solute dilution, and favored chemical and microbiological degradation*.* Kolok et al. (2014) suggested that sediment serves as both a sink and source, equilibrating with VAs during storm events, then slowly releasing them back into water over time, long after the initial pulse of VAs has moved downstream. In contrast, August 2013 produced the lowest detection frequency (40%, Table 5) and median concentration (2.5 ng L^−1^, Fig. 5a) of CTC in the study.

Spatially, CTC detection frequencies were 91–92% for the Lethbridge Northern and St. Mary River IDs and 96% for the remaining IDs in the study (Table 5). Detection frequency of SMZ ranged from 12% (Eastern ID) to zero > LOQ (St. Mary River and United-Mountain View), while TYL was lowest for United-Mountain View (8%), with other IDs somewhat higher (12–23%). For MON, the Taber and Bow River IDs were higher (21–25%) than others (2–11%). The Lethbridge Northern ID had highest detection frequency of ERY (27%), while Western ID was lowest (7%). Overall, across 7 VAs, there was only slight variation in mean detection frequencies, ranging from 31% for United-Mountain View to 36% for Taber.

It was expected that IDs with higher intensive livestock production would show greater detection frequencies of VAs. However, apart from the highest detection frequency of ERY (27%), Lethbridge Northern ID (home to “Feedlot Alley”) had the lowest detection frequencies of CTC (91%) and MON (2%), the second lowest of SMZ (2%), and the third lowest of TYL (13%) (Table 5). Previous studies in the SSRB also failed to draw statistically significant relationships between intensive livestock production and water quality (Johnson et al. 2003; Little et al. 2003). Hyland et al. (2003) and Little et al. (2003) attributed increased bacterial contamination of surface water following precipitation events to aspects other than animal density, e.g., runoff topology of the landscape, and farm-specific practices, such as animal grazing and stream access, and the timing and rate of manure application, factors which likely also apply to VA contamination.

**Fig. 6:**
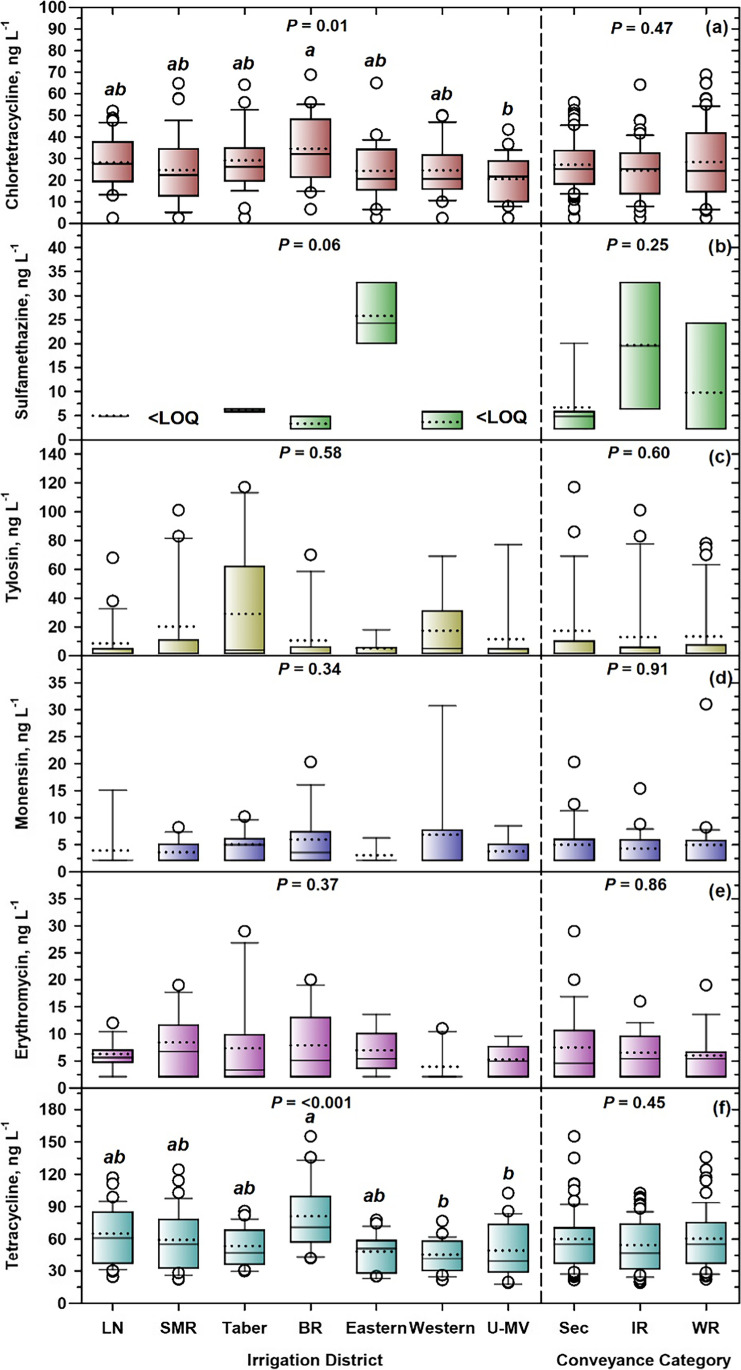
Box plots for concentrations of veterinary antimicrobials in irrigation conveyances: **a** CTC, **b** SMZ, **c** TYL, **d** MON, **e** ERY, and **f** TC, grouped by irrigation district (LN, Lethbridge Northern; SMR, St. Mary River; BR, Bow River; U-MV, United-Mountain View), and conveyance category (Sec, secondary; IR, infrastructure return; WR, watershed return). Within boxes, a solid horizontal line marks the median concentration. A dotted horizontal line marks the mean concentration. The lower boundary of the box indicates the 25th percentile and the upper boundary the 75th percentile. Whiskers (error bars) indicate 10th and 90th percentiles. Circle symbols indicate outliers falling outside the 10th and 90th percentiles. *P* values are presented for Kruskal–Wallis tests on each group of samples. When significant (*P* < 0.05), box plots within groups with different letters have significantly different median concentrations based on Dunn’s test

Irrigation district was significant for CTC (Fig. 6a) and TC (Fig. 6f), but non-significant for SMZ, TYL, MON, and ERY median concentrations (Figs. 6b–e). For CTC, the median concentration in the Bow River ID was significantly greater (32 ng L^−1^) than that in the United-Mountain View IDs (22 ng L^−1^) (Fig. 6a). For TC, there was a similar finding, with the Bow River ID being significantly higher (*x̃* = 73 ng L^−1^) than United-Mountain View (42 ng L^−1^), as well as the Western ID (44 ng L^−1^) (Fig. 6f). As such, the “control” United-Mountain View IDs, chosen to represent less intensive livestock production, with fewer and smaller confined feeding operations, and more extensive cattle grazing, showed significantly lower median concentrations of CTC (by 31%) and TC (by 42%) than the Bow River ID. While this behavior was somewhat anticipated for the United-Mountain View IDs, the Lethbridge Northern ID rather than the Bow River ID was expected to be significantly higher. While the Bow River ID has beef cattle feedlots, their size and density are less than the Lethbridge Northern ID, while dairy and swine operations are also less common. Unfortunately, livestock statistics in Alberta are only available for counties or rural municipalities, rather than IDs, so direct relationships between livestock numbers in an ID and VA concentrations are difficult to generate.

Compared to sampling time and ID, conveyance category had limited influence on detection frequency (Table 5), varying by only 3–5 percentage points for CTC (93–96%), MON (9–12%), and SMZ (1–6%). Across all 7 VAs, however, detection frequency was almost identical (33–34%) for the three conveyance categories. However, even though both TYL and ERY showed slight increases in detection frequencies between secondary conveyances (15%) and watershed returns (21–22%) (Table 5), this did not follow through to median concentrations, with conveyance category non-significant (*P* = 0.25–0.91) for all six VAs (Fig. 6). This finding was somewhat unexpected as watershed returns are natural channels, which collect excess water from irrigation, as well as natural drainage flow (runoff), and occasionally ditch water, or municipal effluent (Charest et al. 2015). Most of the water flow in watershed returns originates from within an ID, and without irrigation, many would be dry in summer. Infrastructure returns are constructed canals at the end of an IDs infrastructure and are therefore generally less influenced by surface runoff (Charest et al. 2015). In southern Alberta, Little et al. (2003) showed that irrigation returns had higher concentrations of dissolved P, total N, and *E. coli*, suggesting an impact of livestock manure. Similarly, Charest et al. (2014) observed a general increase in the concentrations of salts, nutrients, and pathogens from the primary to secondary to return sites, and most water quality parameter concentrations were higher in watershed returns than in infrastructure returns. However, in line with our results, Cessna et al. (2001) showed only minor effects of nutrients from irrigation returns on receiving water bodies. Furthermore, Charest et al. (2015) studied the impact of irrigation returns on rivers and concluded that the effect was negligible.

### Veterinary antimicrobials in a high-intensity agroecosystem

Tetracycline was the only VA with 100% detection frequency in both feedlot and irrigation conveyance samples. Chlortetracycline was a close second with 100% detection frequency in feedlot environs and 94% in irrigation conveyances. Daghrir and Drogui (2013) reported that tetracycline antimicrobials were most widely used globally for veterinary and human therapy. In addition, Mompelat et al. (2009) reported excretion rates of 80–90% for TC, compared with only 5–10% for ERY, while physico-chemical properties of TC defined its hydrophilic character, with high water solubility, and lower octanol–water partition coefficients (*K*_ow_). Also, a relatively low Henry’s constant indicated that TC was weakly lost via volatilization. In addition, dissipation half-live of TC (20–41 days in soil/compost mixture; Li et al. 2010) would also play a role. For four VAs, detection frequencies were substantially lower for irrigation conveyances vs. feedlot samples: 4 vs. 23% for SMZ, 15 vs. 84% for TYL, 10 vs. 94% for MON, and 18 vs. 66% for ERY. Lincomycin was the only VA detected in feedlot environs that was undetectable (< LOQ) in irrigation conveyance water.

As well as manure application, transport pathways to explain the ubiquity of TC, and to a lesser extent CTC in our study, include entry to surface water via runoff, following discharge of catch basin contents onto cropland through irrigation systems. This practice is widely employed, including at Feedlot A (Tymensen et al. 2017), especially if catch basins reach capacity during months of highest precipitation (May–July). In addition, until recently, airborne transport of VAs to downwind aquatic systems was given little consideration (McEachran et al. 2015). In Texas, Sandoz et al. (2018) found a significant relationship between distance to nearest cattle feedyard and MON concentration in wetlands. They pointed out that while aerial VAs transport may be minimal in humid regions with moderate to high rainfall, it was potentially a major pathway in semiarid regions. Southern Alberta is semiarid, with strong chinook winds (> 100 km h^−1^) that may carry particulate matter, and embedded VAs, downwind of feedlots.

Five of six VAs showed significantly (*P* < 0.001) greater median concentrations in feedlot environs than irrigation conveyances: CTC, 277 vs. 25 ng L^−1^; TYL, 42 vs. 2.5 ng L^−1^; MON, 436 vs. 2.5 ng L^−1^, ERY, 9.9 vs. 5.6 ng L^−1^, and TC, 53 vs. 56 ng L^−1^. The exception was SMZ, where there was no significant difference (*P* = 0.92) between feedlot environs (*x̃* = 6 ng L^−1^) and irrigation conveyances (*x̃* = 5.5 ng L^−1^). These VA concentrations were similar to previously reported values from agricultural settings (Bak and Bjorklund 2014; Couperus et al. 2016; Jaimes-Correa et al. 2015; Sandoz et al. 2018). They were several orders of magnitude lower than the minimum inhibitory predicted no-effect concentration (PNEC-MIC), a conservative parameter used for protection against antimicrobial resistance: CTC, 277 vs. 4000 ng L^−1^; TYL, 42 vs. 4000 ng L^−1^; ERY, 9.9 vs. 1000 ng L^−1^; TC, 53 vs. 1000 ng L^−1^ (Bengtsson-Palme and Larsson 2016; Tell et al. 2019).

The only comparative study in Alberta (Forrest et al. 2011) analyzed a total of 247 water samples in 2005–2006 from 23 streams identified as having predominantly agricultural activities in their watersheds. Five VAs common to our study were quantified: CTC, SMZ, MON, LIN, and ERY. Their detection frequencies vs. irrigation conveyances in our study were similar for SMZ (8 vs. 4%) and LIN (1.2% vs. zero), but much lower for CTC (0.4 vs. 94%), somewhat lower for ERY (0.8 vs. 18%), and higher for MON (34 vs. 10%). However, their MDLs were 10 ng L^−1^ for CTC, LIN, and ERY, and 2 ng L^−1^ for SMZ and MON, compared to 2.5 ng L^−1^ in our study. Maximum concentrations reported by Forrest et al. (2011) were higher than corresponding concentrations in our irrigation conveyances for MON (843 vs. 31 ng L^−1^) and LIN (18 ng L^−1^ vs. < LOQ), but lower for CTC (20 vs. 69 ng L^−1^), SMZ (20 vs. 33 ng L^−1^), and ERY (10 vs. 29 ng L^−1^).

Previous research in southern Alberta has quantified CTC, SMZ, and TYL concentrations in runoff (Table 6) from feedlot pen floors (Sura et al. 2015), compost windrows (Sura et al. 2016), and manured cropland (Amarakoon et al. 2014). Concentrations generally declined as the runoff source area moved away from feedlot pen floors. The value of composting as a manure management alternative was evident by large decreases in concentrations from a 2- vs. 21-day-old compost. Once manure was land applied (60 Mg ha^−1^ wet wt.), and especially when soil-incorporated, runoff concentrations fell further, by ~ 2 orders of magnitude compared to feedlot pen floors. Data from the current study (Table 6) shows further declines in CTC and SMZ concentrations in surface water from feedlot catch basins and adjacent creeks. However, there was surprisingly little difference in TYL concentrations between runoff from manured cropland and surface water from irrigation conveyances. There were ~ 5 orders of magnitude difference in concentrations of CTC and SMZ in runoff from feedlot pen floors vs. surface water from irrigation conveyances. For TYL, the difference was ~ 4 orders of magnitude.

**Table 6:** Comparison of mean concentrations of CTC, SMZ, and TYL in runoff and surface water from studies conducted in the South Saskatchewan River Basin

Matrix	Source location	Source details	CTC	SMZ	TYL
			μg L^−1^		
Runoff water	Feedlot pen floors^a^	Bedding area	5260	4570	540
		Non-bedding area	3130	3730	220
	Compost windrows^b^	Day 2^c^	2580	3600	4930
		Day 21^c^	200	980	200
	Manured cropland^d^	Surface applied	59	3.9	0.02
		Soil-incorporated	15	2.6	0.06
Surface water^e^	Feedlot environs	Catch basin	0.82	0.008	0.14
		Retention ponds/wetland	0.39	0.007	0.15
		Creek	0.13	< LOQ	0.009
	Irrigation conveyances	Watershed return	0.028	0.01	0.013
		Infrastructure return	0.024	0.02	0.013
		Secondary	0.027	0.007	0.017

^a^Sura et al. (2015)

^b^Sura et al. (2016)

^c^Start of composting = day 0

^d^Amarakoon et al. (2014)

^e^Present study

While it is important to quantify the presence of VAs occurring in surface waters, it is arguably more critical to determine whether the compounds affect aquatic organisms, especially where diverse classes of VAs are detected simultaneously, exposing organisms and food webs to “antibiotic cocktails” (Danner et al. 2019). While individual concentrations may be low, combined concentrations can result in significant toxicity to aquatic life because of synergistic effects (Grenni et al. 2018). Table S6 shows that overall, 37% of feedlot environ samples showed detection of six target VAs, while 17% showed detection of all seven. However, no creek samples showed detection of all seven target VAs, compared to 22–24% of catch basin and retention ponds/wetland samples, while 13% of creek samples showed detection of six VAs, compared to 43–49% of catch basin and retention ponds/wetland samples. Overall, for irrigation conveyance samples (2014–15 only), the majority of samples (77%) exhibited detections of two VAs, with 21% three VAs, and only 1% four VAs (Table S6). The latter compared with 92% of feedlot environ samples exhibiting four or more VAs. Unlike detection frequency or median concentration parameters, the “antibiotic cocktail” approach provided slight evidence of increased occurrence of VAs in watershed returns (23% showing at least three VAs), compared to secondary sites (18% showing at least three VAs). It is noteworthy that VA concentrations in creeks and irrigation conveyances in our study were several orders of magnitude lower than the 1 μg L^−1^ inhibitory concentration for aquatic organisms (European Medicines Evaluation Agency 2008; United States Food and Drug Administration 1997), a threshold based on retrospective reviews of ecotoxicity data from environmental assessments. These VA concentrations were also several orders of magnitude lower than the PNEC-MIC values (CTC, 4.0 µg L^−1^; TYL, 4.0 µg L^−1^; LIN, 2.0 µg L^−1^; ERY, 1.0 µg L^−1^; TC, 1.0 µg L^−1^ (Bengtsson-Palme and Larsson 2016; Tell et al. 2019).

In surface water in Nebraska, Naderi Beni et al. (2020) detected only one VA (MON), prescribed to confined and grazing livestock at an adjacent farm. Other prescribed VAs (CTC, TYL, SMZ) were undetectable, while non-prescribed VAs (LIN, ERY) were detected. Of the seven VAs in our study, only two (TYL, MON) are used exclusively in veterinary medicine, while the remainder are used in both veterinary and human medicine. The detection of some classes of antimicrobials not used to treat livestock potentially indicated their production by endogenous soil bacteria, e.g., ERY (Schafhauser et al. 2018). In addition, ERY used in human therapy can attach to biosolids at wastewater treatment plants and ultimately reach groundwater after land application (Yan et al. 2014). Biosolids from the City of Lethbridge are applied to local agricultural land and may eventually access irrigation conveyance returns. Thus, surface water contamination originating from veterinary products cannot always be distinguished from that arising from human medicine, as the same antimicrobials may be used throughout the one health continuum (Charuaud et al. 2019). In a rural Indiana stream, Bernot et al. (2013) reported that acetaminophen and caffeine concentrations of human origin were ~ 1 order of magnitude greater than veterinary pharmaceuticals SMZ and LIN.

## Conclusions

We have shown the omnipresence of TC, and to a lesser extent CTC, in surface water of a high-intensity agroecosystem in southern Alberta. Detection frequencies and median concentrations were much lower for SMZ, TYL, and ERY. Monensin detection frequency and concentration were higher in feedlot environs than in irrigation conveyances. Lincomycin was undetectable in irrigation conveyance water.

Our study highlights the challenge of linking the environmental occurrence of VAs to local sources. While VA concentrations may be low, especially in irrigation conveyances, environmental persistence may be a more important consideration in defining strategies for managing VAs in agroecosystems to reduce the risk, and elucidating their roles in antimicrobial resistance. It is evident from our work that research gaps exist and further investigation on environmental fate of VAs is warranted, especially in high-intensity agroecosystems regions such as the South Saskatchewan River Basin.

## Supplementary Information

Below is the link to the electronic supplementary material.

Supplementary file1 (DOCX 41.9 KB)

## Data Availability

Not applicable.
