# Supplementary material for: Veterinary antimicrobials in cattle feedlot environs and irrigation conveyances in a high-intensity agroecosystem in southern Alberta, Canada
Source: Environ Sci Pollut Res Int. 2022 Sep 15;30(5):12235–56. doi: 10.1007/s11356-022-22889-x (PMC9898329; doi:10.1007/s11356-022-22889-x)
Supplement: Supplementary file 1 — Supplementary file1 (DOCX 41.9 KB) [file 11356_2022_22889_MOESM1_ESM.docx]

**Supplementary material**

**Veterinary antimicrobials in cattle feedlot environs and irrigation conveyances in a high- intensity agroecosystem in southern Alberta**

Srinivas Sura ^1*^ • Francis. J. Larney ^2^ • Jollin Charest ^3^ • Tim A. McAllister ^2^ • John V. Headley ^4^ • Allan J. Cessna ^5^

^1^ Agriculture and Agri-Food Canada, Morden Research and Development Centre, 101 Route 100, Morden, MB R6M 1Y5, Canada

^2^ Agriculture and Agri-Food Canada, Lethbridge Research and Development Centre, 5403 1st Avenue S., Lethbridge, AB T1J 4B1, Canada

^3^ Natural Resource Management Branch, Alberta Agriculture, Forestry, and Rural Economic Development, 5401 1st Avenue S., Lethbridge, AB T1J 4V6, Canada

^4^ Environment and Climate Change Canada, National Hydrology Research Centre, 11 Innovation Blvd., Saskatoon, SK S7N 3H5, Canada

^5^ Agriculture and Agri-Food Canada, Saskatoon Research and Development Centre, 107 Science Place, Saskatoon, SK S7N 0X2, Canada

**Table S1: Liquid chromatography mobile phase gradient elution timetable.**

**Table S2: Relative Standard Deviations (RSD, %) values to assess repeatability of the method for all VAs at various concentrations levels.**

Table S3: Precursor ion-product ion (multiple reaction monitoring-MRM transitions), mass spectrometer acquisition parameters, method detection limit (MDL), limit of quantification (LOQ) and extraction recoveries (mean ± standard deviation, SD) for lincomycin, tetracycline, iso-chlortetracycline, sulfamethazine, 13C6-sulfamethazine (internal standard), erythromycin, tylosin, and monensin. Class of each veterinary antimicrobial is included in parenthesis.

**Table S4: Detection frequencies (>LOQ) of seven antimicrobials in water samples from feedlot sampling sites, 2014 to 2016.**

**Table S5. Precipitation amounts associated with irrigation conveyance sampling, 2013**–**15.**

**Table S6 Number of target veterinary antimicrobials detected (>LOQ) in surface water samples from feedlot environs and irrigation conveyances (expressed as percent of total samples) SSRB, 2013–16.**

**Table S1: Liquid chromatography mobile phase gradient elution timetable.**

| Time | Mobile Phase A, % | Mobile Phase B, %) | Flow rate (mL min^-1^) | Curve |
| --- | --- | --- | --- | --- |
| 0:00 | 85.0 | 15.0 | 0.2 | 1 |
| 2:00 | 85.0 | 15.0 | 0.2 | 11 |
| 20:00 | 0.0 | 100.0 | 0.2 | 6 |
| 20:10 | 85.0 | 15.0 | 0.2 | 1 |
| 25:00 | 85.0 | 15.0 | 0.2 | 1 |

**Table S2: Relative Standard Deviations (RSD, %) values to assess repeatability of the method for all VAs at various concentrations levels (*n=5)*.**

| Concentrations (ng mL^-1^) | CTC | SMZ | TYL | MON | LIN | ERY | TC |
| --- | --- | --- | --- | --- | --- | --- | --- |
|  | -------------------------------Relative Standard Deviation (%)------------------------------- | | | | | | |
| 2.5 | 4.1 | 3.9 | 4.1 | 5.1 | 3.9 | 4.6 | 6.1 |
| 5.0 | 4.2 | 5.2 | 3.6 | 4.2 | 3.1 | 2.6 | 5.4 |
| 10.0 | 2.2 | 4.1 | 3.3 | 3.2 | 2.4 | 3.4 | 4.2 |
| 25.0 | 1.9 | 2.4 | 3.1 | 2.5 | 1.5 | 2.6 | 3.1 |
| 50.0 | 2.2 | 2.1 | 1.5 | 2.2 | 1.8 | 2.4 | 2.2 |
| 100.0 | 2.5 | 1.9 | 1.8 | 2.1 | 1.7 | 2.1 | 3.4 |

Table S3: Precursor ion-product ion (multiple reaction monitoring-MRM transitions), mass spectrometer acquisition parameters, method detection limit (MDL), limit of quantification (LOQ) and extraction recoveries (mean ± standard deviation, SD) for lincomycin, tetracycline, iso-chlortetracycline, sulfamethazine, 13C6-sulfamethazine (internal standard), erythromycin, tylosin, and monensin. Class of each veterinary antimicrobial is included in parenthesis.

| Veterinary Antimicrobial  (*Class of compound*) | Parent ion to product ion transitions (m/z) | Cone voltage (v) | Collision energy (ev) | Retention time (min) | Method detection limit, MDL  (ng L^-1^) | Limit of quantification, LOQ  (ng L^-1^) | Recoveries (%, Mean ± SD) | | | |
| --- | --- | --- | --- | --- | --- | --- | --- | --- | --- | --- |
|  |  |  |  |  |  |  | Fortification Level  (10 ng L^-1^) | | Fortification Level  (100 ng L^-1^) | |
|  |  |  |  |  |  |  | 2013^b^ | 2014-16^b^ | 2013 | 2014-16 |
| Lincomycin  (*Lincosaminide*) | 407.2 > 126.0  407.2 > 359.3 | 40.00  40.00 | 28.00  18.00 | 1.74 | 2.5 | 5.0 | 58 ± 16 | 54 ± 12 | 60 ± 14 | 59 ± 10 |
| Tetracycline  (*Tetracycline***)** | 445.0 > 410.0  445.0 > 427.0 | 20.00  20.00 | 20.00  10.00 | 2.10 | 2.5 | 5.0 | - | 105 ± 21 | - | 110 ± 18 |
| *Iso*-Chlortetracycline^a^  (*Tetracycline***)** | 478.9 > 443.9  478.9 > 461.9 | 16.00  16.00 | 22.00  22.00 | 2.43 | 2.5 | 5.0 | 55 ± 9 | 60 ± 10 | 58 ± 6^c^ | 66 ± 8^c^ |
| Sulfamethazine  (*Sulfonamide***)** | 279.2 > 155.7  279.2 > 185.7 | 35.00  30.00 | 18.00  16.00 | 3.32 | 2.5 | 5.0 | 71 ± 21 | 74 ± 18 | 74 ± 18 | 73 ± 17 |
| ^13^C_6_-Sulfamethazine  (*Sulfonamide*) | 285.0 > 186.0  285.0 > 162.0 | 35.00  35.00 | 16.00  18.00 | 3.32 | - | - | - | - | - | - |
| Erythromycin  (*Macrolide***)** | 734.5 > 157.9  734.5 > 576.2 | 30.00  30.00 | 30.00  30.00 | 11.41 | 2.5 | 5.0 | 46 ± 8 | 55 ± 9 | 48 ± 12 | 58 ± 12 |
| Tylosin  (*Macrolide***)** | 916.6 > 174.0  916.6 > 772.4 | 70.00  70.00 | 38.00  32.00 | 11.96 | 2.5 | 5.0 | 82 ± 21 | 89 ± 15 | 88 ± 19 | 91 ± 13 |
| Monensin  (*Ionophore*) | 693.5 > 461.2  693.5 > 675.4 | 85.00  85.00 | 40.00  40.00 | 18.95 | 2.5 | 5.0 | 64 ± 15 | 67 ± 14 | 72 ± 12 | 69 ± 15 |
| ^a^Chlortetracycline irreversibly isomerizes to *iso*-chlortetracycline in water (Cessna et al. 2011) thus iso-chlortetracycline was monitored instead of chlortetracycline.  ^b^WCX, Weak cation exchange cartridge was used for extraction of 2013 samples whereas SAX, Strong anion exchange cartridge was used for extraction of 2014-2016 samples.  ^c^*Iso*-chlortetracycline recoveries were significantly improved (*P* < 0.05) when extracted with SAX cartridges compared to those with WCX while the remaining recoveries were similar. | | | | | | | | | | |

**Table S4: Detection frequencies (>LOQ) of seven antimicrobials in water samples from feedlot sampling sites, 2014 to 2016.**

| **Group** | Samples, *n* | CTC | SMZ | TYL | MON | LIN | ERY | TC | Overall mean^a^ |
| --- | --- | --- | --- | --- | --- | --- | --- | --- | --- |
|  |  | ————————————— **Detection frequency (>5 ng L^-1^), %** ————————————————— | | | | | | | |
| **Sampling site** |  |  |  |  |  |  |  |  |  |
| Feedlot A, Catch basin 1 | 15 | 100 | 40 | 93 | 100 | 100 | 80 | 100 | 88 |
| Feedlot A, Catch basin 2 | 15 | 100 | 33 | 100 | 100 | 93 | 73 | 100 | 86 |
| Feedlot A, Primary retention pond | 16 | 100 | 38 | 94 | 100 | 88 | 81 | 100 | 86 |
| Feedlot A, Constructed wetland | 18 | 100 | 22 | 89 | 100 | 89 | 61 | 100 | 80 |
| Feedlot A, Secondary retention pond | 7 | 100 | 43 | 100 | 100 | 71 | 71 | 100 | 84 |
| Feedlot A, Creek-downstream | 9 | 100 | 0 | 56 | 89 | 44 | 67 | 100 | 65 |
| Feedlot B, Catch basin | 12 | 100 | 17 | 100 | 100 | 25 | 92 | 100 | 76 |
| Feedlot B, Creek-downstream | 11 | 100 | 0 | 73 | 82 | 45 | 27 | 100 | 61 |
| Feedlot B, Creek-upstream | 11 | 100 | 0 | 36 | 64 | 55 | 27 | 100 | 55 |

^a^Based on 7 analyses (CTC, SMZ, TYL, MON, LIN, ERY, TC) of 114 samples (*n* = 7 × 114 = 798).

**Table S5: Precipitation amounts associated with irrigation conveyance sampling, 2013**–**15.**

| Year | Sampling time | Precipitation period | Precipitation, mm |
| --- | --- | --- | --- |
| 2013 | 11–13 June | 24 May–13 June^a^ | 79 |
|  | 27–29 August | 9–29 August | 3 |
| 2014 | 10–12 June | 23 May–12 June | 28 |
|  | 7–10 July | 20 June–10 July | 27 |
|  | 5–7 August | 18 July–7 August | 22 |
|  | 2–4 September | 15 August–4 September | 78 |
| 2015 | 8–11 June | 22 May–11 June | 19 |
|  | 6–9 July | 19 June–9 July | 24 |
|  | 27–30 July | 10–30 July | 34 |
|  | 31 August–3 September | 14 August–3 September | 29 |
| 2013 | All | 1 May–31 July | 199 |
| 2014 | All | 1 May–31 July | 196 |
| 2015 | All | 1 May–31 July | 109 |

^a^21-d period ending on last day of sampling.

**Table S6: Number of target veterinary antimicrobials detected (>LOQ) in surface water samples from feedlot environs and irrigation conveyances (expressed as percent of total samples) SSRB, 2013–16.**

| Sample source | Samples, *n* | **————** Number of veterinary antimicrobials detected **————** | | | | | | |
| --- | --- | --- | --- | --- | --- | --- | --- | --- |
|  |  | 1 | 2 | 3 | 4 | 5 | 6 | 7 |
|  |  | **——————————** % samples **———————————** | | | | | | |
| **Feedlot environs** | | |  |  |  |  |  |  |
| All | 114 | 0 | 3 | 5 | 13 | 25 | 37 | 17 |
|  |  |  |  |  |  |  |  |  |
| Catch basins | 42 | 0 | 0 | 0 | 5 | 28 | 43 | 24 |
| Retention pond/wetland | 41 | 0 | 0 | 0 | 12 | 17 | 49 | 22 |
| Creek | 31 | 0 | 10 | 19 | 26 | 32 | 13 | 0 |
|  |  | |  |  |  |  |  |  |
| **Irrigation conveyances (2014–15 only)^a^** | | | |  |  |  |  |  |
| All | 190 | 1 | 77 | 21 | 1 | 0 | 0 | 0 |
|  |  |  |  |  |  |  |  |  |
| Secondary | 72 | 1 | 81 | 18 | 0 | 0 | 0 | 0 |
| Infrastructure return | 62 | 2 | 74 | 21 | 3 | 0 | 0 | 0 |
| Watershed return | 56 | 0 | 77 | 23 | 0 | 0 | 0 | 0 |

^a^2013 not included because TC not analyzed.
